# The anatomy of a crushing bite: The specialised cranial mechanics of a giant extinct kangaroo

**DOI:** 10.1371/journal.pone.0221287

**Published:** 2019-09-11

**Authors:** D. Rex Mitchell

**Affiliations:** 1 Zoology Division, School of Environmental and Rural Sciences, University of New England, Armidale, New South Wales, Australia; 2 Department of Anthropology, University of Arkansas, Fayetteville, Arkansas, United States of America; Monash University, AUSTRALIA

## Abstract

The Sthenurinae were a diverse subfamily of short-faced kangaroos that arose in the Miocene and diversified during the Pliocene and Pleistocene. Many species possessed skull morphologies that were relatively structurally reinforced with bone, suggesting that they were adapted to incorporate particularly resistant foods into their diets. However, the functional roles of many unique, robust features of the sthenurine cranium are not yet clearly defined. Here, the finite element method is applied to conduct a comprehensive analysis of unilateral biting along the cheek tooth battery of a well-represented sthenurine, *Simosthenurus occidentalis*. The results are compared with those of an extant species considered to be of most similar ecology and cranial proportions to this species, the koala (*Phascolarctos cinereus*). The simulations reveal that the cranium of *S*. *occidentalis* could produce and withstand comparatively high forces during unilateral biting. Its greatly expanded zygomatic arches potentially housed enlarged zygomaticomandibularis muscles, shown here to reduce the risk of dislocation of the temporomandibular joint during biting with the rear of a broad, extensive cheek tooth row. This may also be a function of the zygomaticomandibularis in the giant panda (*Ailuropoda melanoleuca*), another species known to exhibit an enlarged zygomatic arch and hypertrophy of this muscle. Furthermore, the expanded frontal plates of the *S*. *occidentalis* cranium form broad arches of bone with the braincase and deepened maxillae that each extend from the anterior tooth rows to their opposing jaw joints. These arches are demonstrated here to be a key feature in resisting high torsional forces during unilateral premolar biting on large, resistant food items. This supports the notion that *S*. *occidentalis* fed thick, lignified vegetation directly to the cheek teeth in a similar manner to that described for the giant panda when crushing mature bamboo culms.

## Introduction

Feeding ecology and skull morphology have an intimate association. The distinct mechanical properties of some foods can encourage morphological specialisations to improve their procurement and efficient processing. Phylogenetically distinct taxa sometimes express similar responses to such dietary challenges [1–6], mediated via a combination of evolutionary convergence and phylogenetic constraints [1]. In this manner, convergent morphology can be seen to represent convergent feeding ecology and behaviour, leading to more robust conclusions that link form and function. In the mammalian skull, the inclusions of particularly resistant foods into the dietary range are often accompanied by convergent aspects of the dentition, craniomandibular bone morphology, and jaw adductor musculature across a range of taxa [3, 7–16]. Yet, trade-offs are inevitable between performance and resource allocation during the evolutionary development of such features.

Additional bony buttressing of the craniofacial region helps to resist high forces experienced across the cranium during the breakdown of mechanically resistant foods [17–18]. However, bone is a metabolically expensive material to produce. The degree of bone reinforcement should therefore reflect the minimum required to successfully perform the most strenuous actions demanded [1,19]; alongside the accommodation of safety factors [20]. A taxon known for their particularly robust craniofacial morphology are the extinct short-faced kangaroos of the marsupial subfamily Sthenurinae. Many sthenurine species possessed brachycephalic, broad and deepened crania, with enlarged zygomatic arches and expanded frontal bones [21]. However, there remains little quantitative support for the roles of these features in the biomechanics of feeding. Here, three-dimensional finite element analysis [22] is employed to address the biomechanical significance of the robust features that are present in the cranium of one well-represented species of short-faced kangaroo, *Simosthenurus occidentalis*.

The sthenurines arose during the Miocene and diversified during the Pliocene/Pleistocene to encompass six genera and 26 species [21,23–24]. Some sthenurines are considered to have been gigantic compared to the largest extant kangaroos that reach ~90 kg [25] and included the largest kangaroo species to ever exist, *Procoptodon goliah* (est. 224–244 kg [26]). The subfamily is united by nine cranial synapomorphies, making it the most clearly defined suprageneric taxon within the Macropodidae [21]. Of these nine cranial features, over half are directly associated with the feeding apparatus, encompassing aspects of the dentition, the temporomandibular joint (TMJ), and the mandible. The distinctive cranial and postcranial morphology of this taxon may have represented the occupation of ecological roles, and associated behaviours, no longer observed among extant Australian herbivores [21,27–28]. Members of the Simosthenurinii tribe (comprised of the genera *Archaeosimos*, *Simosthenurus* and *Procoptodon*), for which cranial specimens are known, had particularly brachycephalic and deep crania with broad zygomatic arches and wide frontal plates. Raven and Gregory [29] suggested that the large sthenurine jaws, and massive molars bearing thick longitudinal crests with complex plications, indicated a diet focussed on coarser shrubs, while Ride ([30], p.54) considered the robust cranial morphology of *Procoptodon* to represent “a secondary adaptation to the most heavy type of browsing”. More recently, the robust cranial morphology of *P*. *goliah* has been discussed with regards to its capacity for generating high masticatory forces, and has been identified as a chenopod browse specialist, via a combination of dental microwear and stable-isotope analyses [31].

**Fig 1:**
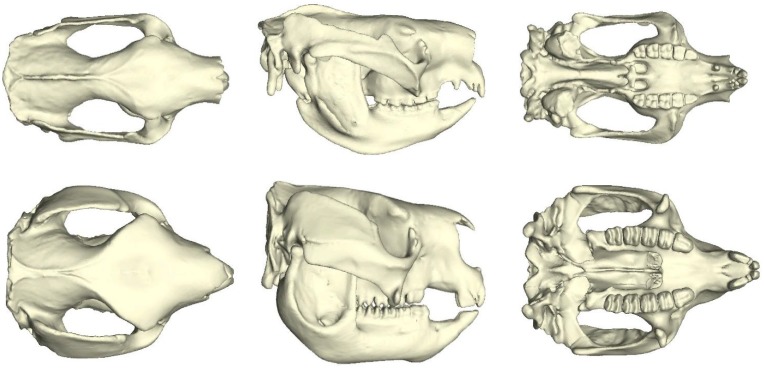
A comparison between the skulls of the koala (top) and *Simosthenurus occidentalis* (bottom). Views from left to right are dorsal, lateral, and ventral.

*Simosthenurus occidentalis* was a medium-sized sthenurine but giant compared to extant kangaroos, with an estimated average body mass of ~118 kg [26]. Fossil evidence for this species exists across the southern states of Australia [21], where it possibly persisted until ~42 ka [32–33]. The likelihood of this species being a browser has long been assumed [21,34] and recently supported via comparative shape analysis and biomechanical approaches, where it was found to have similar craniofacial proportions and palatodental arrangements to the koala (*Phascolarctos cinereus*) [35]. However, several features that differ from the koala, including its overall much larger size, relatively large check teeth, and generally deepened cranium, suggest that it could consume much tougher, bulky plant matter (Fig 1).

The impressive cheek tooth battery of *S*. *occidentalis* extends back beyond that of the koala, below the neurocranium and closer to the jaw joints (Fig 1). This may be problematic if the rear molars are engaged in biting, as the closer the rear teeth are to the jaw joint, the greater the probability of dislocation [36–38]. During such instances, larger, more resistant food items may become the functional fulcrum of the jaw lever system [39] resulting in tensile (distractive) forces, and potential injury, at the TMJ. Although this can be often be mediated by adjusting the amount of muscle force that is applied from the opposing side (balancing-side) to the bite [36], the notable proximity of the rear molars to the TMJs in *S*. *occidentalis* suggests a potentially precarious system. A specialised masticatory muscle anatomy may, therefore, have been required in order to reduce such risks if resistant food items were to come between the rear molars of *S*. *occidentalis* during mastication.

Despite the observed positive allometry between body mass and zygomatic arch breadth (dorsoventral length) among extant kangaroos and their relatives [40], the arches of *S*. *occidentalis* are relatively enormous across this aspect (Fig 1). In both placental and marsupial mammals, virtually the entire medial surface of the zygomatic arch represents the fleshy origin of the zygomaticomandibularis (ZM) muscle [41–42]. Muscle force is proportional to muscle cross-sectional area [43] and an increase in these dimensions of the zygomatic arch would therefore permit an increase to the cross-sectional area of the ZM. This muscle has been suggested to pull the mandible transversely during the grinding of foods in other placental mammals [6,44]; however, the lateral phase of the chewing cycle in extant kangaroos tends to be carried out by the biting side (working-side) superficial masseter and working-side medial pterygoid [45]; and Warburton [42] suggested that the function of this muscle is to elevate and retract the mandible in kangaroos. The medial surface of the zygomatic arch in the *S*. *occidentalis* cranium offers a potential surface area of ZM muscle origin rivalling that of the temporalis muscle origin. A hypertrophied ZM may have provided some protection against TMJ dislocation during biting towards the rear of the tooth row. Thus, the first hypothesis of this study asks whether an increase to the muscle force of the ZM influenced molar biting performance and TMJ integrity in this species.

If *S*. *occidentalis* was indeed capable of consuming particularly tough vegetation, its wide cranium likely permitted greater muscle mass and associated bite forces [8]; and these would, in turn, be expected to produce powerful axial twisting (torsion) of the cranium during unilateral biting on larger, resistant objects [19]. Therefore, the second hypothesis of this study asks whether the cranium of *S*. *occidentalis* is better adapted to resist such twisting forces. Results for *S*. *occidentalis* are compared with those of the koala for both hypotheses. Previous simulations of these two species have shown comparable mechanical efficiency and bone deformation during incisor biting [35]. Therefore, differences in their mechanical performance during unilateral biting simulations may be attributable to their respective degrees of specialisation to withstanding torsional forces.

## Materials and methods

The finite element models (FEMs) for both species used here are from Mitchell & Wroe [35] and follow the same construction protocols. Surface meshes were created from computed tomography (CT) data in Mimics (Materialise v. 19). The *S*. *occidentalis* model is a reconstructed cranium of Victoria Fossil Cave SAMA P16648. The specimen was missing some of the left-side zygomatic arch and portion of the occipital plate and these were replaced through mirroring and superimposition. The missing coronoid processes of the mandible were replaced using those of a swamp wallaby (*Wallabia bicolor*). As these were only required to estimate a centroid for the temporalis muscle, the potential differences in temporalis muscle vector orientation attributable to differing coronoid process morphology would likely have a negligible influence on the results for these hypotheses (see [35]).

The surface meshes were converted to volume meshes (FEMs) using 3-Matic (Materialise v. 10) and composed of ~1.7 million three-dimensional tetrahedral elements. The two FEMs were then imported to Strand7 (v. 2.4.4). All elements were allocated the average material properties of mammalian bone (Young’s modulus: E = 20 GPa; Poisson’s ratio: v = 0.3) [46]. Homogeneous and isotropic properties were considered adequate, as the comparative nature of this study is focussed on the influence of cranial shape and associated musculature on mechanical performance [47–49]. Therefore, the results of these models can be used to identify relative differences in shape and structure but should not be considered to represent in vivo bite forces or stress magnitudes [50].

The masticatory musculature was partitioned into the seven divisions identified for macropods [42]: the deep masseter (MD), intermediate masseter (MI), superficial masseter (MS), lateral pterygoid (PL), medial pterygoid (PM), temporalis (T), and zygomaticomandibularis (ZM). The total muscle force of a red-necked wallaby (*Macropus rufogriseus*) was obtained from Mitchell, et al. [40], for an arbitrary reference value to scale to each model. This data has also been applied to these two models previously [35]. As an interest of this study was on the influence of muscle proportions on resultant force magnitudes, this total muscle force was scaled to cranial volume using a 2/3 power rule [47] to ensure that both models were experiencing similar input forces for their size, but then partitioned into their respective taxonomic proportions. For the koala, the muscle proportions were obtained from Sharp [45] and the MS and MI were pooled and treated as a single unit to align with the muscle origin delineations of Davison and Young [51]. Since no actual muscle proportions can be accurately obtained from fossil material for *S*. *occidentalis*, these values had to be estimated. Despite the koala being more similar in proposed ecology and craniofacial proportions to *S*. *occidentalis* than other macropods [35], there are unique modifications in the pterygoid musculature of the koala that result in a masticatory system more akin to some placental herbivores [52], making these proportions less appropriate to represent *S*. *occidentalis*. Thus, muscle proportions of a more closely related browser, Lumholtz’s tree-kangaroo (*Dendrolagus lumholtzi*), were used for *S*. *occidentalis*. Only proportions for major muscle divisions for the tree-kangaroo were available [42]. Subdivisions of these muscle complexes were considered negligible compared to major divisions, so the proportions for the red-necked wallaby were maintained, within the major divisions of the tree-kangaroo. To test the influence of the ZM muscle size, the force of this muscle was increased fourfold, on a separate identical FEM of *S*. *occidentalis*, to align more closely with the temporalis, as suggested by the comparable surface areas of the muscle origins for these muscles (Fig 1). This adjustment resulted in similar muscle proportions between the temporalis and ZM. All muscle forces and proportions are presented in Table 1.

**Table 1:** Muscle forces (N) and proportions of total muscle force (%) applied to each finite element model: The koala (*Phascolarctos cinereus*), *Simosthenurus occidentalis* with tree-kangaroo muscle proportions, and the second *S*. *occidentalis* model with and adjusted (adj.) ZM muscle.

	MD	MI	MS	PL	PM	T	ZM	Total
*P*. *cinereus* forces (N)	73.63	NA	174.04	26.78	93.71	234.28	66.94	669.38
*P*. *c*. muscle proportions (%)	11.00	NA	26.00	4.00	14.00	35.00	10.00	100.00
*S*. *occidentalis* (N)	200.37	313.85	284.92	200.43	200.43	770.67	215.02	2185.68
*S*. *o* (%)	9.17	14.36	13.04	9.35	8.98	35.26	9.84	100.00
*S*. *occidentalis* (adj.) (N)	200.37	313.85	284.92	200.43	200.43	770.67	860.06	2830.73
*S*. *o*. (adj.) (%)	7.08	11.09	10.07	7.22	6.93	27.23	30.38	100.00

The gape angle was set to 5 degrees from the TMJ to the incisors using Geomagic Studio (v. 2014). The respective forces for each muscle were then applied to their origins using BoneLoad [53]. This software directs the muscle forces towards the centroid of their respective insertions on the mandible. A separate BoneLoad file was created for four percentages of balancing-side muscle force: 100%, 50%, 25% and 10% of the working-side muscle force. This permitted interpolation of the entire range of reaction forces at the TMJs for all proportions of balancing-side muscle recruitment.

To simulate a unilateral bite at each cheek tooth, the balancing-side TMJ was restrained against translation along the lateral, dorsoventral and anteroposterior axis, while the working-side TMJ was only restrained in the dorsoventral and anteroposterior axis [17,54–55]. Simulations were carried out at the premolar (P3) and all molars (M1–M4) along the tooth row by restraining a single node against dorsoventral translation at the centre of the occlusal surface for each tooth of interest. To reinforce the restrained regions against artificial stress singularities at the individual restrained nodes, each TMJ and restrained tooth was tessellated with stiff beams to distribute stress more evenly [56]. In total, 60 simulations were carried out: five teeth, at four values of balancing-side force recruitment, across three models.

To examine the first hypothesis, bite reaction forces were obtained from the occlusal plane for each tooth at the restrained node, while joint reaction forces were obtained from their respective restrained nodes by creating a Cartesian coordinate system. This system represents a “triangle of support” formed by the bite point and working-side and balancing-side TMJs and reaction forces were obtained from the axis perpendicular to the plane [17,54]. The respective directions of the reaction forces indicated whether they were compressive (+) or tensile (-) and if both TMJs were found to be experiencing compressive forces, the bite was balanced, without risk of dislocation. Although the majority of unilateral biting and mastication likely occurs towards the middle-anterior of the cheek tooth row, focus of interpretations was drawn to M4 biting as a proxy for TMJ integrity during specific muscle loadings, since the M4 represents the region of the functional tooth row most at risk of causing injury when utilised and, if an M4 bite is balanced, then all other teeth anterior to the M4 will also produce successful bites under those conditions. Mechanical efficiency was then calculated by dividing bite reaction force by total muscle force applied. Mechanical efficiency is consistent across all muscle loadings for each tooth [57] and is, therefore, presented for each tooth only, rather than also including the results for each loading case.

The second hypothesis was examined by comparing stress distributions of unilateral P3 bite simulations, with 100% balancing-side muscle force recruited. Heat maps of von Mises stress enabled visual comparison of biting performance between the models. von Mises stress was used because the main interest of this hypothesis was the torsional forces experienced across the crania and stress is a representation of force per unit of area [22].

## Results

**Fig 2:**
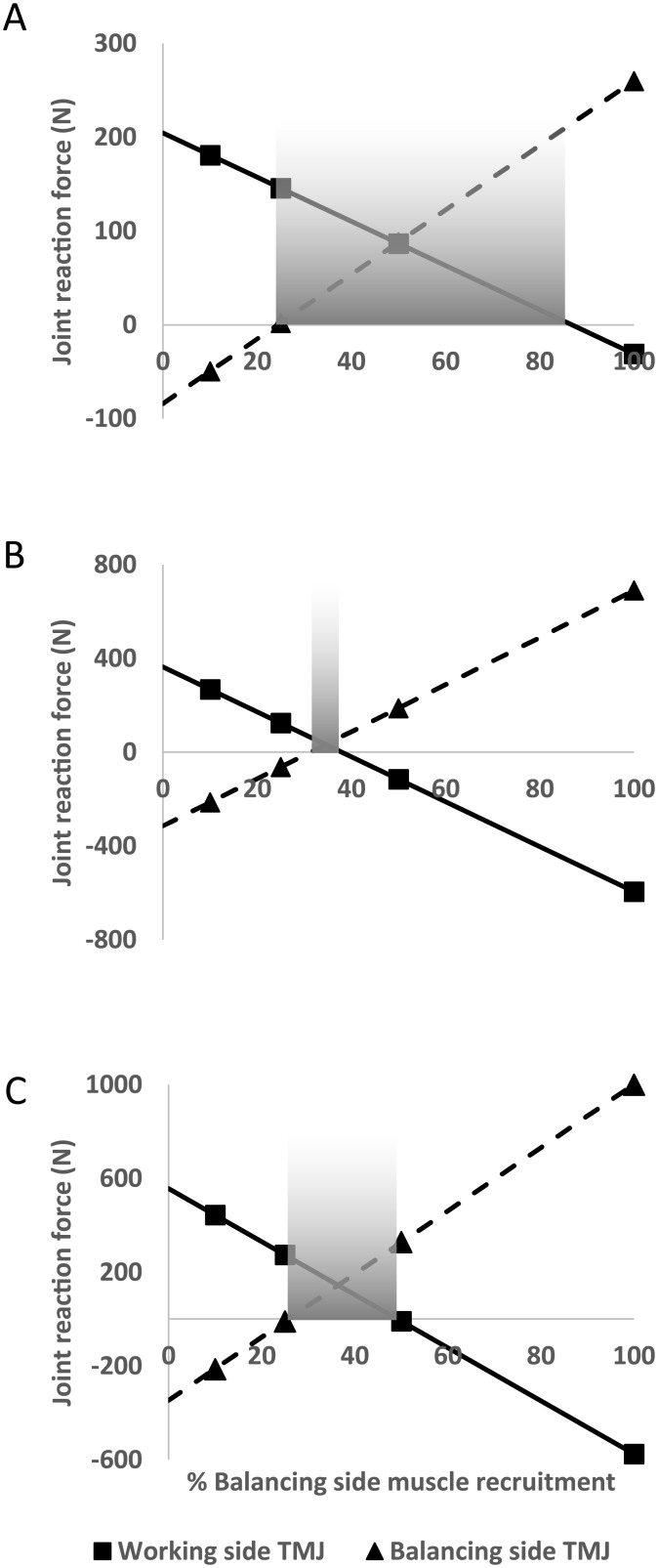
Plots of working and balancing TMJ reaction forces during M4 biting across the entire range of balancing-side muscle recruitment, interpolated from the four measurements (triangles and squares). Negative values indicate tensile forces. Shaded regions indicate potential ranges of muscle recruitment that result in a balanced triangle of support. (A) A broad range of balancing-side muscle recruitment is observed for M4 biting in the koala model (B) the *Simosthenurus occidentalis* model with tree-kangaroo muscle proportions has almost no range of balance at the M4 (6.70%) (C) when the ZM muscle of *S*. *occidentalis* is increased in size, the range of balancing-side muscle recruitment that results in a balanced triangle of support is increased (23.46%).

The simulations suggest that the koala has a particularly well-balanced feeding apparatus (Table 2). A balanced bite, with compressive forces at all points of the triangle of support, was possible with maximum muscle force recruitment along the entire tooth row up until the M3 molar. Beyond this point, the M4 molar is in balance with an application of 50% balancing-side muscle force. By contrast, the triangle of support of *S*. *occidentalis* is only in balance with maximum muscle force up to the M2 molar. The M3 molar is then in balance at 50% of balancing-side muscle force. No loading scenarios resulted in a balanced bite at the M4 molar for either the tree-kangaroo muscle proportions or when the *S*. *occidentalis* ZM muscle force was increased. However, the tensile forces of the TMJs are markedly lower at both 50% and 25% of balancing-side muscle recruitment (Table 2), suggesting a greater range of balanced scenarios. The interpolated values for working-side and balancing-side joint forces during these M4 bites indicate that this is the case (Fig 2). A balanced system is possible at the M4 (and, therefore, for the entire functional tooth row) of the koala with balancing-side muscle forces of 24.40% to 86.84% (range: 62.44%) (Fig 2A). By contrast, a balanced rear molar bite for *S*. *occidentalis* with tree-kangaroo muscle proportions is virtually unattainable, with a balanced triangle only occurring at the M4 between a balancing-side muscle force of 31.30% to 38.00% (range: 6.7%) (Fig 2B). However, the addition of increased ZM muscle force results in a balanced system between 25.75% and 49.21% (range: 23.46%) (Fig 2C).

**Table 2:** Reaction forces at each biting tooth and TMJ during each muscle loading. BMF = balancing-side muscle force recruited, TMF = total muscle force applied, WS = working-side TMJ, BS = balancing-side TMJ. Tensile forces (-) represented by bold-italicised values.

	*Phascolarctos cinereus*				*Simosthenurus occidentalis*				*Simosthenurus occidentalis* (adj.)			
BMF	100%	50%	25%	10%	100%	50%	25%	10%	100%	50%	25%	10%
**TMF**	1338.76	1004.07	836.73	736.32	4371.36	3278.52	2732.10	2404.25	5661.45	4246.09	3538.41	3113.8
**P3**	422.26	315.78	262.54	230.59	1542.04	1153.67	959.49	842.98	1857.53	1387.52	1152.51	1011.50
**WS**	155.02	231.27	269.39	292.26	501.46	749.45	873.44	947.84	781.60	1083.86	1235.00	1325.68
**BS**	334.91	140.00	42.55	***-15*.*92***	1089.52	448.10	127.38	***-65*.*04***	1512.40	637.87	200.61	***-61*.*75***
**M1**	468.58	350.41	291.33	255.89	1843.18	1378.97	1146.86	1007.60	2220.28	1658.48	1377.58	1209.03
**WS**	123.62	207.16	248.94	274.00	292.64	589.89	738.52	827.69	525.20	883.65	1062.87	1170.41
**BS**	326.56	134.07	37.83	***-19*.*92***	1075.80	440.00	122.09	***-68*.*65***	1493.98	630.65	198.98	***-60*.*02***
**M2**	531.97	397.83	330.75	290.51	2220.19	1661.03	1381.45	1213.70	2674.43	2591.23	1659.35	1456.34
**WS**	78.97	172.68	219.53	247.65	84.30	428.53	600.65	703.92	268.82	379.80	887.34	1011.05
**BS**	317.06	127.72	33.06	***-23*.*74***	1016.20	399.84	91.67	***-93*.*24***	1419.15	499.34	166.14	***-84*.*46***
**M3**	625.86	468.03	389.12	341.78	2879.82	2154.52	1791.87	1574.29	3469.01	2591.23	2152.35	1889.01
**WS**	26.44	131.66	184.27	215.83	***-218*.*92***	189.59	393.85	516.40	***-106*.*14***	466.17	622.76	768.54
**BS**	292.07	110.39	19.55	***-34*.*96***	907.24	328.85	39.65	***-133*.*86***	1281.30	497.93	108.36	***-126*.*23***
**M4**	739.80	553.24	459.97	404.00	4108.41	3073.69	2556.33	2245.91	4948.96	3696.71	3070.58	2694.91
**WS**	***-30*.*99***	86.76	145.64	180.96	***-594*.*72***	***-115*.*32***	124.38	268.20	***-575*.*21***	***-8*.*94***	274.19	444.07
**BS**	260.08	88.07	2.06	***-49*.*54***	689.90	187.83	***-63*.*21***	***-213*.*83***	1006.64	328.76	***-10*.*18***	***-213*.*55***

**Fig 3:**
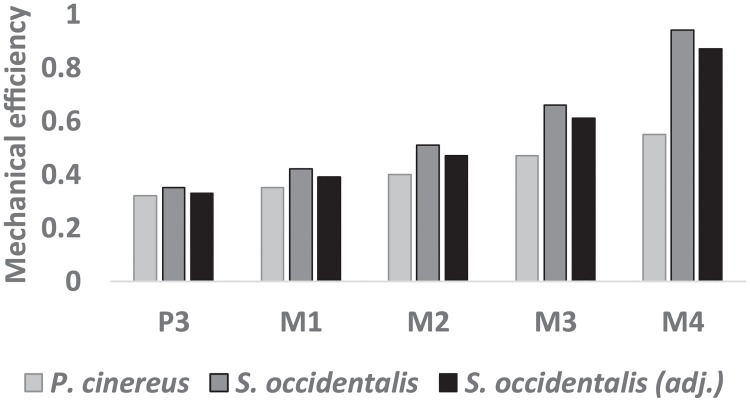
Mechanical efficiency (bite reaction force/total muscle force) at each biting tooth.

Both the koala and *S*. *occidentalis* models display similar mechanical efficiency at the P3, M1, and M2, although *S*. *occidentalis* always has greater values (Fig 3). The differences become more apparent from the M3, as *S*. *occidentalis* demonstrates much greater mechanical efficiency, resulting in a high of 0.94 at the M4 molar, as opposed to 0.55 in the koala. The adjusted ZM muscle slightly decreases the mechanical efficiency for *S*. *occidentalis*; however, it maintains a value of 0.87 at the M4 molar under these loadings.

**Fig 4:**
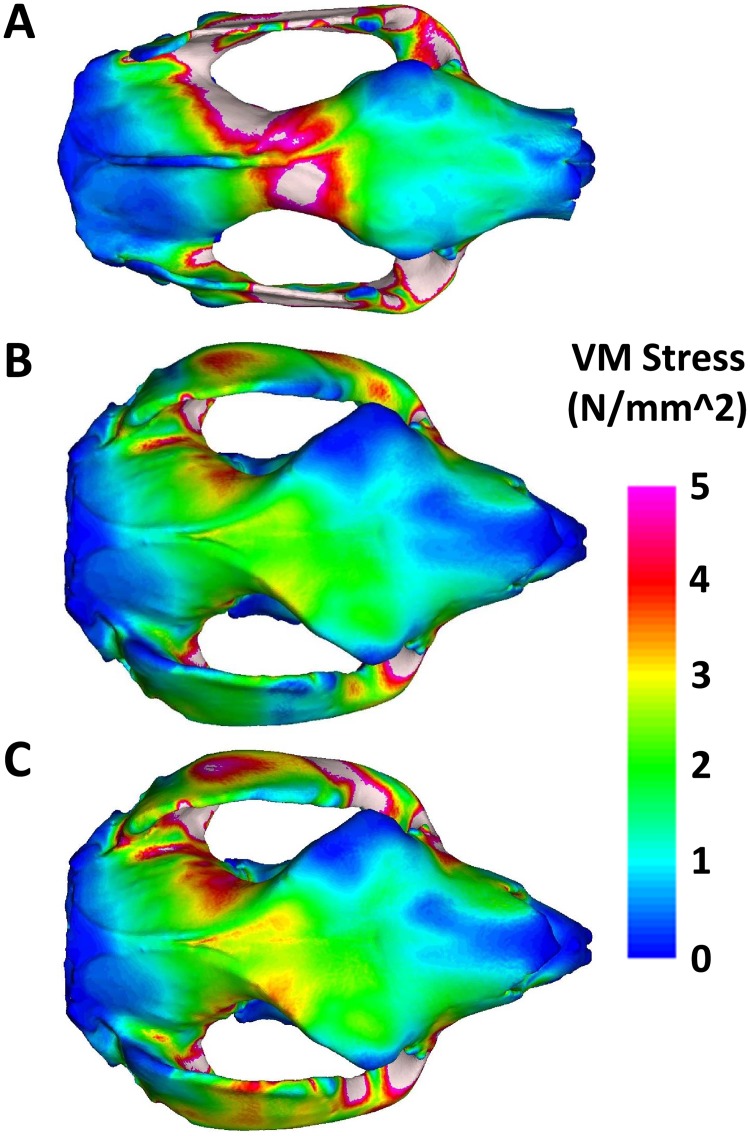
Finite element models depicting Von Mises stress magnitudes during a unilateral premolar bite in (A) the koala, (B) *Simosthenurus occidentalis* with tree-kangaroo muscle proportions, and (C) *S*. *occidentalis* with tree-kangaroo muscle proportions and enlarged ZM muscle.

The FEMs constrained at the P3 premolar with 100% balancing-side muscle force demonstrate that visibly greater stress magnitudes are experienced in the koala model, extending from the balancing-side TMJ, across the braincase, towards the anterior neurocranium and the biting P3 tooth (Fig 4A). Stress is maximised at the anterior of the neurocranium in this model. By contrast, stress is visibly lower along this axis in the *S*. *occidentalis* model (Fig 4B) and stress remains less apparent than the koala even with the addition of the increased ZM muscle forces (Fig 4C).

## Discussion

This study aimed to identify the functional significance of several robust features in the *S*. *occidentalis* cranium. Quantitative support is found for the hypothesis that its dorsoventrally expanded zygomatic arches housed hypertrophied ZM muscles, demonstrated here to act as a balancing agent that minimises distractive forces of the TMJ when the rear molars are utilised for biting. Furthermore, the *S*. *occidentalis* model more effectively resists torsional forces during unilateral biting than the koala model, supporting the second hypothesis. Therefore, both hypotheses are supported by the simulations carried out here and suggest that *S*. *occidentalis* was more capable than the koala of processing larger, more resistant vegetation, both in absolute and relative terms.

**Fig 5:**
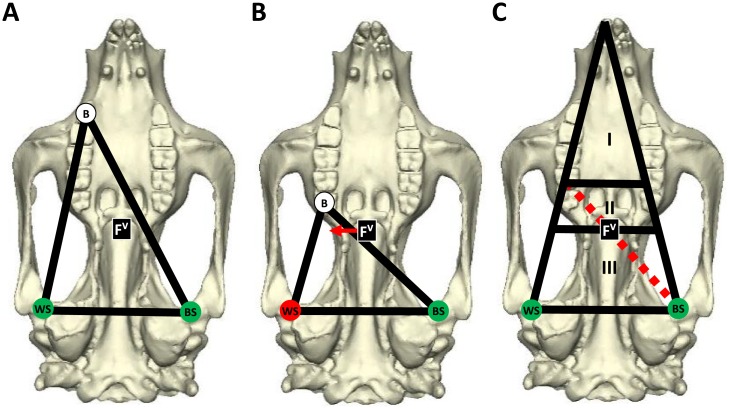
The constrained lever model of jaw biomechanics, as depicted on a koala cranium. (A) The triangle of support is formed from the biting tooth “B”, the working-side TMJ “WS”, and the balancing-side TMJ “BS”. The muscle resultant force vector “F^V^” is centred during full recruitment of all adductor muscles. (B) The F^V^ must be located within the triangle of support in order to maintain compressive forces at the working-side TMJ. Thus, biting at the posterior dentition can result in distractive forces at this joint unless the F^V^ is shifted back within the triangle of support. This is achieved by reducing the balancing-side muscle forces applied. (C) The three regions of balance in the mammalian jaw: Region I extends from the bite point in line with the F^V^ and balancing-side TMJ to all dentition anterior to this point; Region II exists between the posterior limit of Region I and the line of mediolateral F^V^ intersection; Region III commences posterior to the mediolateral line of F^V^ intersection and represents the edentulous portion of the cranium anterior to the TMJs.

The results of the first hypothesis are largely explained by the predictions of the constrained lever model of feeding biomechanics [36,38,58]. This model predicts the risk of dislocation at the jaw joint during mastication, by examining the magnitude and direction of the reaction forces experienced at the three points of the triangle of support. The simultaneous action of all masticatory adductor muscles can be reduced to a single resultant force vector (F^V^) [8,36,59]. The line of action of the F^V^ is typically located just posterior to the tooth row [60–61] and must pass within the triangle of support in order to produce compressive forces at all three points of the triangle [36,38,58] (Fig 5A). Such bites can be achieved via full muscle recruitment; however, biting at the posterior molars can move the triangle of support to the side of the F^V^, which results in distractive forces, and potential dislocation, at the working-side TMJ [36,38,58]. In such instances, the F^V^ can be shifted back within the triangle by reducing the muscle forces of the balancing-side (Fig 5B), thus restoring compressive forces to all points of contact and balancing the bite. The jaw mechanism can therefore be divided into three regions defined by these conditions (Fig 5C): Region I represents the extent of the jaw for which full muscle recruitment on both sides of the cranium can be utilised. Across Region II, balancing-side muscle force must be reduced to prevent distractive forces at the working-side TMJ. Region III represents all locations posterior to the F^V^ and teeth are not expected to be present here, since all bites will potentially result in injury [36,38,58,62–63].

The precarious nature of rear molar biting observed in the *S*. *occidentalis* simulations is borne from this relationship between Region II and Region III. Jaw adductor musculature is often positioned further forward on the faces of mammalian species that must bite harder, because a larger ratio of effort arm/load arm increases mechanical efficiency [40,64–65]. A prediction from the constrained lever model is that no teeth should be positioned within Region III [58,66]. Yet, anteriorly positioned adductor musculature produces a more anteriorly positioned F^V^. Thus, if a given species possesses relatively anteriorly positioned adductor muscles, this should result in either a tooth row that is reduced in length or positioned more anteriorly, or rear molars that are either greatly reduced or missing entirely, in order to position the tooth row within Region I and Region II [36–38].

Both the koala and *S*. *occidentalis* crania possess a masseteric process located further forward than most other marsupial herbivores they have been compared with [35], indicating an anteriorly extended origin of the masseter. The koala follows the predictions of the model, with a relatively small, anteriorly positioned tooth row (Fig 1); however, as mentioned earlier, the robust molar battery of *S*. *occidentalis* would appear to be at odds with these predictions. The simulations conducted here support the hypothesis that the enormous zygomatic arches of *S*. *occidentalis* may have supported hypertrophied ZM muscles. The joint reaction forces indicated that the *S*. *occidentalis* model was virtually incapable of biting with the rear molars, with muscle proportions of a contemporary tree-kangaroo, without experiencing distractive forces at the TMJs. These conditions would render the rear of the functional tooth row useless during mastication due to the risk of injury. However, an increase in the ZM muscle force provided a corresponding increase in the range of balancing-side muscle forces that achieved balanced scenarios, from 6.70% to 23.46% (Fig 2B and 2C). Thus, the additional ZM force moved the F^V^ posteriorly, bringing the M4 out of Region III, into Region II, and enabled the inclusion of the rear of the cheek tooth battery in the breakdown of foods while maintaining remarkably high mechanical efficiency (Fig 3).

A larger ZM may have been present early in sthenurine evolution. The banded hare-wallaby (*Lagostrophus fasciatus*) is the closest extant relative of the sthenurines [67–68] and has a relatively larger ZM than other kangaroos and wallabies [42], suggesting a possible morphological precursor to this adaptive trajectory. The location of the ZM is ideal for balancing hard bites involving the most posterior teeth, as the insertion is located just anterior to the mandibular condyle, and yet posterior to the tooth row [41–42]. The shorter nature of its fibres is noted by Ride [30] and since muscle force is proportional to muscle length [42], this muscle may have provided relatively stronger forces. Moreover, since the orientation of the ZM fibres become increasingly vertical as the mandible lowers [69], this balancing role of the ZM was possibly compounded during wider gapes that may have occurred when crushing particularly large, bulky items, for example. These results, when viewed in light of the constrained lever model, evidence the proposed importance of balancing-side muscles in sthenurine mastication [21,27] but do not preclude the possibility that the ZM also contributed to lateral movements of the mandible [6,44], as they potentially played an important role in grinding actions as well. If so, a large ZM muscle in *S*. *occidentalis* may have served functions towards both jaw mobility and jaw joint stability, as suggested for the M. depressor mandibulae muscle in cynodont therapsids [39].

The highly dynamic nature of mastication requires constant adjustments to applied muscle forces, as there is an ongoing possibility of intermittent distractive forces acting on the working-side TMJ and the likelihood of this increases with more posterior bites [58]. As the bite point on the tooth row moves posteriorly, the joint reaction forces decrease while the bite force increases [38,58] (Table 2). In addition, lower joint forces occur when muscles are positioned further forward [57], and, in the case of *S*. *occidentalis*, teeth positioned further back as well. Thus, M4 molar biting in the adjusted *S*. *occidentalis* model expressed very high mechanical efficiency (0.87), but the joint reaction forces are extremely low during these bites. For example, with a balancing-side muscle force of ~36% applied, both joint reaction forces are estimated to have compressive forces of 144.75 N (Fig 2C), or around 2.6% of the total muscle force. A 23.46% leeway for balanced bites with an enlarged ZM is a narrower range than the koala and, with these low joint reaction forces, the dynamic nature of mastication could still have put the TMJ at risk of injury if the M4 molars were used. This may be a function of the enlarged ectoglenoid process, another of the nine synapomorphies that unite the sthenurines [21]. This mesial expansion of the posterior jugal borders the glenoid fossa and represents the attachment of the lateral mandibular ligament [21]. An enlargement of this ligament would have likely reinforced the TMJ and helped to resist dislocation of the TMJ during dynamic crushing at the M4 [70]. Furthermore, other actions, such as contraction of the digastric muscle, locking of the incisors, and support from the robust postglenoid process may also have lessened the risk of TMJ injury [21].

**Fig 6:**
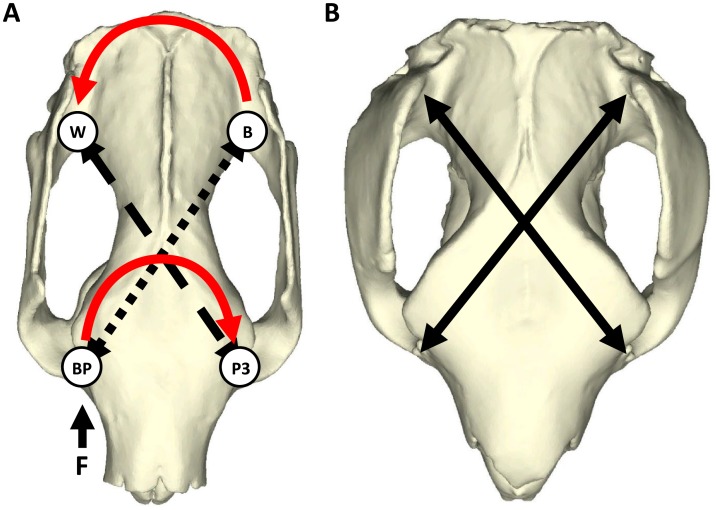
Torsional arcs during a unilateral bite. (A) A koala cranium performing a unilateral premolar bite. As the biting premolar “BP” receives a reaction force “F” from a hard food item, the anterior cranium rotates clockwise along the axis between “BP” and the opposite premolar “P3”. At the same time, the balancing TMJ “B” rotates anticlockwise along the line to the working-side TMJ “W” in response to the higher joint reaction forces at “B”. This produces compressive forces (short dashes) along the surface of the cranium between “BP” and “B”; and tensile forces (long dashes) between “P3” and “W”. (B) The enlarged frontal plates of *Simosthenurus occidentalis* follow these helical arcs of compression and tension (unbroken lines) by expanding from the anterior brain case to the supraorbital tuberosity above the cheek tooth row.

The simulations revealed that the koala model experiences concentrations of stress along a line extending from the balancing-side TMJ to the region of the biting premolar (Fig 4A). These stress distributions can be attributed to the forces experienced from torsion. During unilateral biting, the compressive bite reaction force at the tooth rotates the cranium around the longitudinal axis in one direction, while the greater joint reaction force at the balancing-side TMJ rotates the rear of the skull in an opposing direction [19] (Fig 6A). These asymmetrical forces create an axial twisting of the cranium that generates compressive forces along a helical arc from the biting tooth, dorsally across the surface of the skull, to the balancing-side TMJ (Fig 6A) [19,60]. The stress distribution observed across the dorsal surface of the koala model follows this arc. By contrast, the *S*. *occidentalis* simulations resulted in a similar distribution of stress, but visibly lower magnitudes than the koala (Fig 4B), thus supporting the second hypothesis. Furthermore, this remained relatively consistent with the addition of over 600 N of muscle force from the enlarged ZM (Fig 4C), further demonstrating that the *S*. *occidentalis* model is remarkably resistant to torsion.

If additional buttressing of bone is required to resist torsion during unilateral biting, it is expected to be concentrated along the helices of maximum stress and strain [19], particularly around the weak junction of the anterior neurocranium and posterior splanchnocranium near to the rear of the orbits [60,71], as found in the koala model. A distinctive synapomorphic feature of the sthenurine cranium is the lateral expansion of the frontal bones [21,35]. Prideaux [21] suggested that these may help to orient the temporalis muscle more vertically, protect the eyes during browsing, and reinforce the postorbital ligament that protects the eye from the flexing of the surrounding muscles during chewing. While these may certainly represent advantages of this morphology, the frontal bones also appear to correspond with the helical arcs of torsion, from the anterior cheek tooth (P3-M1) region to the balancing-side TMJs (Fig 6B). This is achieved via the lateral expansion of the frontals from the anterior neurocranium to the supraorbital tuberosity. Stress is equal to force per unit area [22] and an increase in the area of bone therefore results in a decrease in stress for a given input force. The broad frontal plates and deep maxillae of the *S*. *occidentalis* cranium increase the total area of bone along the torsional helix, potentially decreasing maximum point-stress. In this manner, torsional stress can be observed dispersed at lower magnitudes across this expansive region on the dorsal surface of the *S*. *occidentalis* model during a premolar bite simulation (Fig 4B and 4C). The large zygomatic arches may also have provided additional bracing, thereby serving multiple functions for hard unilateral biting. Thus, the simulations performed in this study suggest that *S*. *occidentalis* had a cranial morphology with the capacity for delivering crushing bites to particularly resistant vegetation.

The efficiency by which S. *occidentalis* could obtain tough browse vegetation via the incisors has been supported previously [35]. However, the toughest tissues may have required greater leverage. *Simosthenurus occidentalis* had robust premolars that were frequently longer than all other cheek teeth [21] (Fig 1). Robust tooth morphology is associated with the processing of resistant foods [3] and the large premolars in this species may therefore have been a focal point for crushing such items. Previous studies of sthenurines have suggested that there may have been occasions during foraging that the incisors were bypassed, and vegetation was fed directly to the cheek teeth [21,31]. The results of this study provide quantitative support for such actions in *S*. *occidentalis* and suggest potentially similar feeding behaviours to the giant panda (*Ailuropoda melanoleuca*). This secondarily herbivorous carnivoran utilises post-canine dentition for the majority of the preparation and processing of mature bamboo culms, the most resistant plant tissues in its diet and mostly consumed during less productive, colder seasons [72]. The largest diameter culms (up to 38mm [73]) are cracked between the molars and peeled to expose the interior pith [74]. The peeled culm is then placed crosswise into the cheek tooth row, where it is bitten off and chewed [73]. Given the extensive bony reinforcement shown here to resist torsional stresses arising from the anterior cheek tooth row, it is entirely possible that *S*. *occidentalis* grasped browse vegetation with its powerful forearms and fed larger items directly to the enlarged, molarised premolar in this manner when necessary [21,27–28,31]. The fractured material would then be passed to the large, well-developed molar battery for further crushing. Such behaviours are also commonly observed in the koala when feeding on *Eucalyptus* leaves [51] and *S*. *occidentalis* may have extended these actions to particularly thick and tough plant tissues.

The giant panda has many other cranial features convergently similar to *S*. *occidentalis*, which provide further grounds for inferring potentially similar ecology and behaviour between these two species. These include a foreshortened and deepened skull, an anterior extension of the masseter muscle, broadening of the cheek teeth with molarisation of the premolars, a TMJ located high above the occlusal plane, a lateral extension of the zygomatic arches, an expansion of the frontal region, and, notably, a dorsoventral expansion of the zygomatic arches relative to other bears (Ursidae) [1,6,12,14]. This expansion of the zygomatic arch is known to accommodate the largest ZM muscle found across all Carnivora, lending further support to the first hypothesis of this study [73]. Furthermore, as in *S*. *occidentalis* (Fig 1), the enlarged cheek tooth rows of the giant panda extend behind the mandibular ramus [75], suggesting similarly high mechanical efficiency and potential TMJ distraction if utilising the rear molars during mastication. Therefore, an enlarged ZM may also serve a similar function in balancing a precarious triangle of support during bites at the posterior tooth row in the giant panda.

## Conclusion

The simulations carried out here suggest that the cranial morphology of *S*. *occidentalis* was well-adapted to manage the mechanical demands of generating and resisting high bite forces, supporting previous suggestions that this species was capable of processing thick, resistant forage to a degree not utilised by extant Australian herbivores. Exploitation of bulky, more fibrous browse, such as mature leaves, larger stems, and branches of shrubs and trees, was likely an effective strategy during times when higher quality forage was scarce. This was achieved via modifications to the dentition, bone structure, and jaw adductor muscles. While the koala exhibits a well-balanced triangle of support by following the predictions of the constrained lever model, *S*. *occidentalis* appears to have circumvented this predicted functional constraint by expanding the ZM muscle to maintain a greater probability of TMJ balance whilst converting much more muscle force to bite force along its large cheek tooth row. Furthermore, the expanded frontal plates of *S*. *occidentalis* are shown here to align with the brain case and deepened maxillae to form a broad arch from the anterior cheek teeth to the balancing-side TMJ, capable of supporting high stresses arising from torsion. Both the dorsoventrally expanded zygomatic arch and expanded frontal bones of *S*. *occidentalis* have therefore demonstrated unique functional roles in the delivery of crushing bites. An enlarged ZM muscle may serve a similar function in the giant panda, a species suggested here to potentially share ecomorphological attributes with *S*. *occidentalis*.
